# Invasion of intestinal cells by *Staphylococcus warneri*, a member of the human gut microbiota

**DOI:** 10.1186/s13099-022-00528-7

**Published:** 2023-01-27

**Authors:** Robin Louail, Franklin Florin, Sophie Bernard, Jean-Baptiste Michaud, Jonathan Breton, Najate Achamrah, Marie-Pierre Tavolacci, Moïse Coëffier, David Ribet

**Affiliations:** 1 Univ Rouen Normandie, INSERM, ADEN UMR1073, Nutrition, inflammation and microbiota-gut-brain axis, 76000 Rouen, France; 2 Univ Rouen Normandie, PRIMACEN, HeRacLeS INSERM US51 CNRS UAR2026, 76000 Rouen, France; 3 CHU Rouen, Department of Nutrition, 76000 Rouen, France; 4 CHU Rouen, CIC-CRB 1404, 76000 Rouen, France; 5 INSERM UMR1073 – Université de Rouen, UFR Santé, 22 Boulevard Gambetta, 76183 Rouen Cedex, France

**Keywords:** Host–bacteria interactions, Gut microbiota, Gut microbiome, Coagulase-negative staphylococci, *Staphylococcus warneri*, Intestinal epithelium, Pathogenic potential, Internalization, Intracellular bacteria

## Abstract

Coagulase negative staphylococci (CoNS) are a heterogeneous group of bacteria that colonize different types of human epithelia. These bacteria have a highly variable pathogenic potential ranging from avirulent species to major nosocomial pathogens. *Staphylococcus warneri* is a CoNS species considered to be nonpathogenic. Here, we identify that *S. warneri* is a natural member of both human and mouse gut microbiota. In addition, we demonstrate that this bacterium is able to get internalized into human cells. We show that *S. warneri* efficiently invades several human cell types and, more specifically, intestinal epithelial cells, using actin-dependent mechanisms. In contrast to *bona fide* pathogens, *S. warneri* does not actively replicate within intestinal cells or resist killing by macrophages. Together, our results highlight that bacteria from the human gut microbiota that are not associated with a high pathogenic potential, can actively invade intestinal cells and may, in this way, impact intestinal physiology.

## Background

Human intestinal cells live in close contact with trillions of bacteria [1]. Most of these bacteria thrive in the intestinal lumen under physiological conditions and do not engage in direct physical contact with host cells. In contrast to commensal or symbiotic bacteria, “professional” pathogens may adhere to intestinal cells and eventually get internalized in these cells. Internalization into nonphagocytic cells is a strategy shared by several bacterial pathogens such as *Listeria monocytogenes*, *Salmonella typhimurium* or *Shigella flexneri*. Indeed, adopting an intracellular lifestyle provides various advantages: bacteria become inaccessible to humoral attacks, antimicrobial peptides or even antibiotics; they avoid shear stress-induced clearance and they gain access to a potentially wide range of intracytoplasmic nutrients [2]. These bacteria may finally cross the cell membrane again to egress and infect neighboring cells or get access to deeper tissues [2, 3].

Bacteria–host interactions in the intestine are however more complex than this dichotomy between “good” and “bad” bacteria. Indeed, some gut bacterial species may either act as commensals/symbionts under normal conditions or promote disease when conditions in the intestinal environment are altered. The “pathogenic potential” of bacteria from the gut microbiota is thus highly variable and context-dependent [4, 5].

Coagulase negative staphylococci (CoNS) are a heterogeneous group of bacteria that colonize different types of human epithelia and have been detected both on the skin and in the gut microbiota [6, 7]. These bacteria have a highly variable pathogenic potential. *Staphylococcus epidermidis*, for example, is a major nosocomial pathogen able to invade human cells [6, 8–11]. *S. warneri,* in contrast, is a CoNS species that has only rarely been associated with infections and its ability to invade epithelial cells has not yet been characterized [6]. *S. warneri* is a well-established member of the human skin microbiota. This bacterium has also been detected in the human gut microbiota, but mainly in the case of young children and premature infants [12–17]. The presence of *S. warneri* in the gut microbiota of adults is, in contrast, poorly documented.

In this study, we quantified the prevalence of *S. warneri* in the gut microbiota of human adults, evaluated the ability of this bacterium to get internalized into intestinal cells and discussed the pathogenic potential of this member of the gut microbiota.

## Results

### *S. warneri* is naturally present in the gut microbiota of mice and humans

**Fig. 1:**
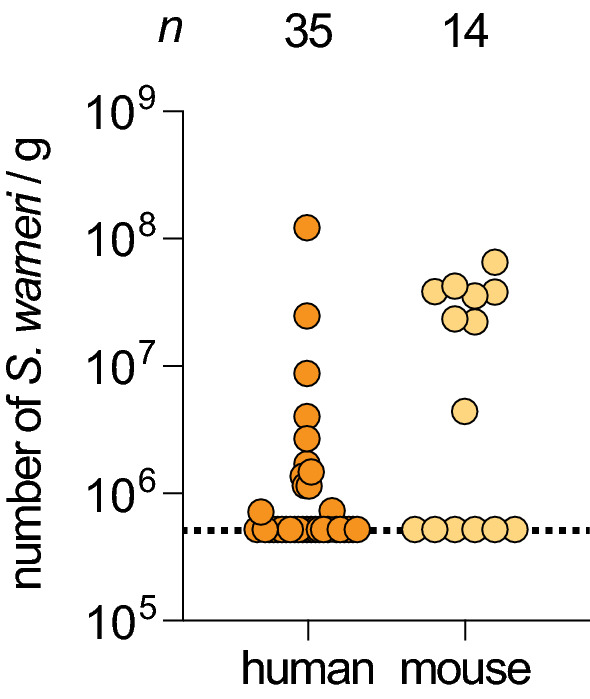
*S. warneri* is a member of the human and mouse gut microbiota. Quantification of *S. warneri* in human fecal samples and mouse cecal contents (detection threshold, 5 × 10^5^ bacteria/g; dashed line). The number (*n*) of individuals or animals analyzed is indicated above the graph

To assess whether *S. warneri* is naturally present in the gut microbiota of humans and mice, we designed specific primers targeting the *S. warneri sodA* gene, which encodes a manganese-dependent superoxide dismutase. This gene exhibits higher sequence divergence than the 16S rRNA gene and thus constitutes a better target to discriminate CoNS species [18]. We used these primers to quantify the number of *S. warneri* by performing quantitative PCR on DNA extracted from pathogen-free murine cecal contents or from fecal samples of healthy individuals. The specificity of these primers was validated by sequencing the amplified PCR products. The total amounts of Eubacteria in these samples were quantified in parallel. In humans, we detected *S. warneri* in 34% of healthy individuals with a mean abundance of 1.3 × 10^7^ bacteria/g of feces (corresponding to ~ 0.08% of the total Eubacteria) (Fig. 1). No significant differences were observed between males and females. No significant correlations could be observed between the fecal abundance of *S. warneri* and the age or the body mass index (BMI) of individuals. In mice, we detected *S. warneri* in the cecal microbiota of 57% of the animals in abundances similar to those observed in humans (3.2 × 10^7^ *S. warneri*/g of cecal content, i.e., ~ 0.06% of the total Eubacteria) (Fig. 1). Together, these results demonstrate that *S. warneri* is a natural member of both mouse and human gut microbiota.

### *S. warneri* can invade human Caco2 cells

**Fig. 2:**
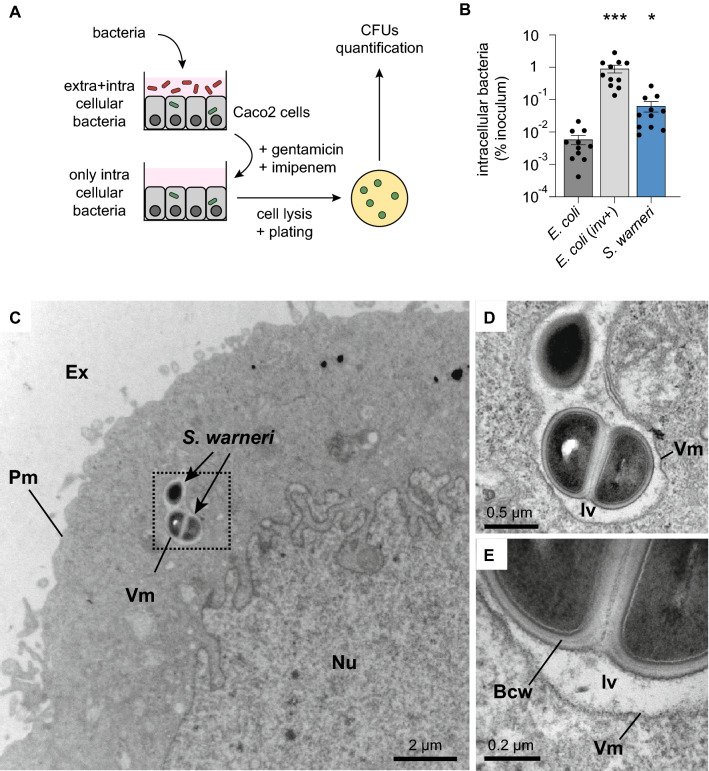
*S. warneri* can invade Caco2 cells. **A** Description of the gentamicin/imipenem-protection assay used to quantify internalization efficiency in Caco2 cells. **B** Quantification of the intracellular bacteria, expressed as the percentage of bacteria initially coincubated with Caco2 cells (% of inoculum) (mean ± s.e.m.; *n* = 11; *P < 0.05; ***P < 0.001 vs. *E. coli*; Friedman test with Dunn’s correction). **C**–**E**, Electron microscopy observations of *S. warneri* internalized in Caco2 cells (Bcw: bacterial cell wall; Ex: extracellular medium; Iv: internalization vacuole; Nu: nucleus; Pm: plasma membrane; Vm: vacuolar membrane)

To evaluate the ability of *S. warneri* to invade intestinal cells, we performed gentamicin/imipenem-protection assays [19] (Fig. 2A). For this, *S. warneri* grown in BHI broth in the stationary-phase were added to Caco2 cells with an MOI (multiplicity of infection) of 100. After 1 h of incubation at 37 °C, extracellular bacteria were eliminated by extensively washing the cells and the addition of a mix of gentamicin and imipenem to the cell culture medium. These two antibiotics do not efficiently cross eukaryotic membranes and thus do not kill intracellular bacteria. The Caco2 cells were then lysed, and cell lysates, containing potential intracellular bacteria, were spread on BHI agar plates. The number of CFUs (colony-forming units) obtained after one day of incubation at 37 °C were then quantified to estimate the number of bacteria that were internalized by Caco2 cells (Fig. 2A). We used as a negative control *E. coli* (K12), which is considered as a noninvasive species. We used as a positive control an *E. coli* strain transformed with an expression vector encoding the *Y. pseudotuberculosis* invasin gene, which confers the ability to efficiently invade nonphagocytic cells (*E. coli/*inv^+^) [20]. Quantification of the CFUs obtained after the gentamicin/imipenem protection assays showed that *S. warneri* was able to invade Caco2 cells, with an internalization efficiency significantly higher than that of *E. coli* (~ 11 times higher) (Fig. 2B).

To confirm these results, we observed Caco2 cells coincubated for 2 h with *S. warneri* by electron microscopy. We found several examples of *S. warneri* internalized in Caco2 cells (Fig. 2C–E). All detected bacteria were localized in internalization vacuoles. We could not detect any bacteria free in the cytosol. These results confirm the ability of *S. warneri* to get internalized into intestinal cells and suggest that this bacterium does not quickly escape from its internalization vacuole, as has been observed for pathogenic bacteria such as *L. monocytogenes* and *S. flexneri* [21].

**Fig. 3:**
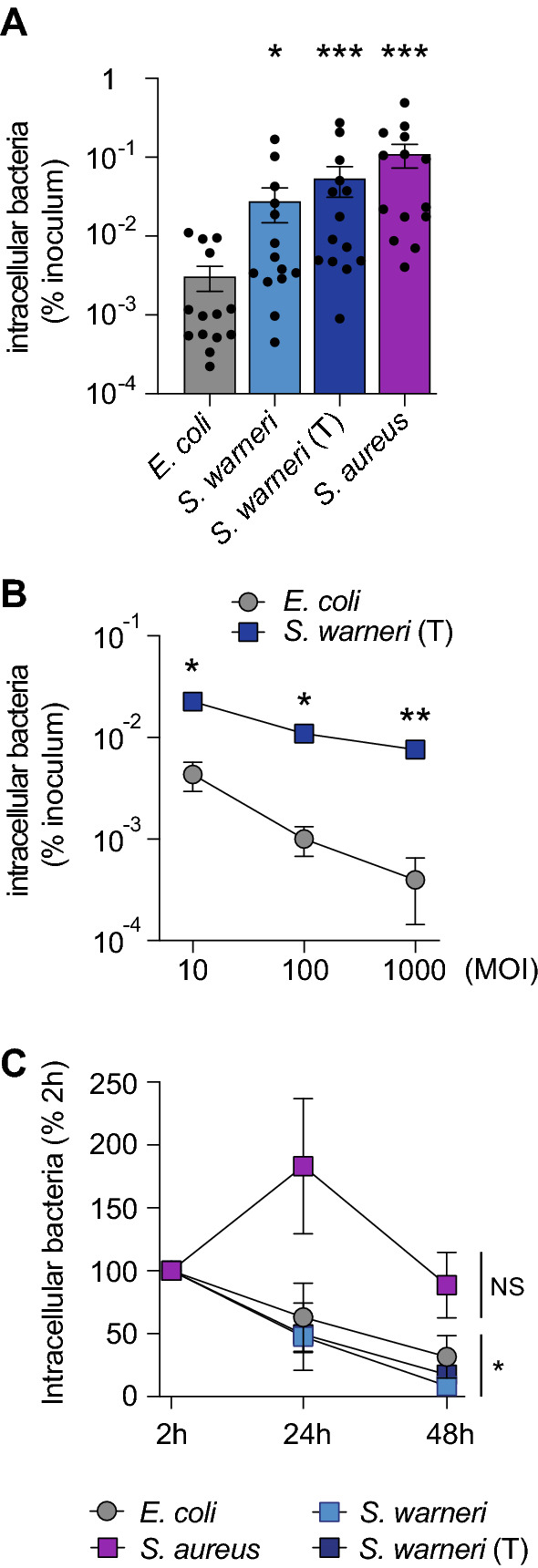
Internalization efficiency and survival of *S. warneri* in nonphagocytic cells. **A** Quantification of intracellular bacteria in Caco2 cells (% of inoculum) (mean ± s.e.m.; *n* = 14; *P < 0.05; ***P < 0.001 vs. *E. coli*; Friedman test with Dunn’s correction). **B** Quantification of intracellular bacteria (% of inoculum) (mean ± s.e.m.; *n* = 3–4; *P < 0.05; **P < 0.01 vs. *E. coli*; 2-way ANOVA with Sidak’s correction). **C** Quantification of intracellular bacteria, expressed as the percentage of intracellular bacteria recovered at 2 h (mean ± s.e.m.; *n* = 6; NS: not significant; *P < 0.05 vs. 2 h; 2-way ANOVA with Sidak’s correction)

To complete our set of results, which were obtained with an intestinal strain of *S. warneri*, we evaluated the internalization efficiency of the *S. warneri* type strain AW25^T^ (isolated from human skin; [22]). We also quantified the internalization efficiency of the bacterial pathogen *Staphylococcus aureus* (RN4220 strain) in parallel [23]. As previously observed with the intestinal *S. warneri* strain, the *S. warneri* AW25^T^ type strain is able to invade Caco2 cells, with an internalization efficiency significantly higher than that of *E. coli* (~ 17 times higher) (Fig. 3A)*.* As expected, *S. aureus* (RN4220 strain) also efficiently invaded Caco2 cells, with an internalization efficiency ~ 36 times higher than that of *E. coli* (Fig. 3A). These results highlight that the invasive properties of *S. warneri* are shared between strains isolated from different niches.

We further evaluated the internalization capacity of the *S. warneri* AW25^T^ type strain in Caco2 cells at different MOIs (10, 100 or 1000). We observed that at each MOI, the internalization efficiency of *S. warneri* was significantly higher than the one of *E. coli* (Fig. 3B).

### Survival of intracellular *S. warneri* in phagocytic and nonphagocytic cell lines

Once internalized, bacteria may either be killed by host cells or survive and eventually proliferate. We evaluated the survival of *S. warneri* in intestinal cells by monitoring the number of intracellular bacteria one or two days after internalization by Caco2 cells. We observed that the number of intracellular *S. warneri* gradually decreased in Caco2 cells, with approximately 50% of the bacteria recovered at 24 h and less than 20% at 48 h (Fig. 3C). In contrast, we showed that the number of intracellular *S. aureus* did not decrease 48 h after internalization (Fig. 3C). These results suggest that *S. warneri* does not actively replicate inside intestinal cells after internalization.

Invading pathogens that cross the intestinal barrier can be phagocytosed and killed by macrophages, unless the phagocytosed bacteria express virulence factors that allow their survival in these immune cells. To determine whether *S. warneri* could resist killing by macrophages, we incubated this bacterium with a murine macrophage cell line (RAW264.7 cells). We observed a large decrease in the number of intracellular *S. warneri,* with only 5.1 ± 4.0% of the bacteria left at 24 h compared to that at 2 h. These results indicate that *S. warneri* is not able to resist killing by macrophages.

### *S. warneri* can invade both intestinal and nonintestinal human cells

**Fig. 4:**
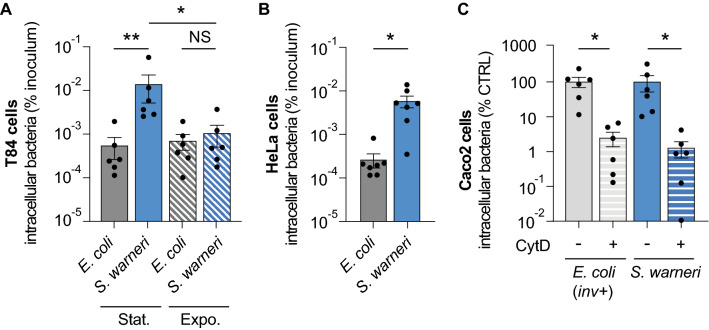
*S. warneri* invades both intestinal and nonintestinal cells using actin-dependent mechanisms. **A** Quantification of the intracellular bacteria in T84 cells after growth in the stationary (stat.) or exponential (expo.) phases (% of inoculum) (mean ± s.e.m.; *n* = 6; *P < 0.05; **P < 0.01; two-tailed Mann‒Whitney test). **B** Quantification of the intracellular bacteria in HeLa cells (% of inoculum) (mean ± s.e.m.; *n* = 7; *P < 0.05; two-tailed Wilcoxon test). **C** Quantification of bacterial internalisation by Caco2 cells pretreated or not with Cytochalasin D (CytD), expressed as a percentage of the control conditions (no CytD) (mean ± s.e.m.; *n* = 6; *P < 0.05; two-tailed Wilcoxon test)

We quantified the internalization of *S. warneri* in two additional human epithelial cell lines: T84 cells, another type of intestinal cell line, and HeLa, nonintestinal cells. We observed significant internalization of *S. warneri* in these cells in comparison to *E. coli* (~ 25 × and ~ 22 × higher than *E. coli* in T84 and HeLa cells, respectively; Fig. 4A and B). Together, these results demonstrate that *S. warneri* is able to invade several nonphagocytic human cell lines in vitro.

We then compared the internalization efficiency of *S. warneri* grown either in the stationary phase or in the exponential phase. Interestingly, we observed a significantly higher internalization of *S. warneri* only for bacteria grown in the stationary phase (Fig. 4A), suggesting that the bacterial factors mediating *S. warneri* internalization are expressed specifically during this growth phase.

### *S. warneri* internalization is actin dependent

The majority of professional intestinal pathogens use actin-based mechanisms to mediate their internalization into nonphagocytic cells [24]. To determine whether *S. warneri* internalization requires host cell actin remodeling, we pretreated Caco2 cells with cytochalasin D, a mycotoxin that inhibits actin polymerization. As expected, we observed that the internalization of *E. coli/*inv^+^ was inhibited by cytochalasin D, since *Y. pseudotuberculosis* invasin-mediated entry is actin-dependent [25] (Fig. 4C). In the case of *S. warneri*, we also observed a large decrease in internalization efficiency by cells treated with cytochalasin D, demonstrating that this CoNS species uses actin-dependent internalization mechanisms to invade host cells (Fig. 4C).

## Discussion

In this study, we highlight that *S. warneri* is a natural member of the human adult gut microbiota with a prevalence of ~ 34% in a population of healthy individuals. Interestingly, we also detected *S. warneri* in the mouse gut microbiota, thereby confirming previous observations of this bacterium in fecal samples from other mammals [26–30]. Together, these results suggest that *S. warneri* is a common member of mammal gut microbiota.

We further demonstrate that *S. warneri* can get internalized into human cells. Using antibiotic-protection assays, we showed that *S. warneri* can efficiently invade several human cell types and, more specifically, intestinal epithelial cells using actin-dependent mechanisms. One limitation of our in vitro invasion assays is the absence of a mucus layer, which, in vivo, keeps intestinal bacteria distant from the epithelium. Even though this mucus layer may preclude *S. warneri* from reaching intestinal cells in vivo under normal conditions, specific conditions such as inflammation, a local decrease in mucus thickness or dysbiosis and proliferation of mucus-degrading intestinal bacteria may facilitate *S. warneri* access to intestinal epithelial cells, eventually leading to their internalization.

The ability of *Staphylococcus* species to invade human cells has most often been studied with *S. aureus*, which can invade various cell lines such as endothelial cells, epithelial cells, keratinocytes and osteoblasts [31]. This host cells invasion allows *S. aureus* to escape host humoral immune responses, avoid shear stress-induced clearance and evade antibiotic activities. *S. aureus* survival in nonphagocytic cells thus facilitates bacterial persistence and promotes chronic infections. Internalization of *Staphylococcus* non-*aureus* species in human cells has also been reported for *Staphylococcus lugdunensis* [11, 32, 33]*, Staphylococcus saprophyticus* [34, 35], *Staphylococcus pseudintermedius* [36], *Staphylococcus delphini* [37] and *S. epidermidis* [8–11], which have been classically considered as opportunistic pathogens [6]. Pathogenic CoNS species are responsible for foreign body-related infections as well as infections in preterm newborns [6]. Colonization of the skin and mucosa by these bacteria may constitute an important source of endogenous infections. These bacteria can indeed switch between the nonpathological colonization of body surfaces to infections and uncontrolled replication owing to decreases in host immune surveillance (for example in immunocompromised individuals) or breaches in the skin or mucosal barriers (for example during the insertion of catheters or implantation of orthopedic devices). Some clinical cases of bloodstream infections by *S. epidermidis* in adults or in preterm neonates showed that genetically similar *S. epidermidis* strains were present in the gut of these patients, which may thus constitute the source of infection [38, 39]. In contrast to *S. epidermidis*, only a few cases of *S. warneri* infections have been reported, suggesting its lower pathogenic potential. Our results, which show that *S. warneri* can invade enterocytes, raise the question of the consequences of these internalization events on intestinal physiology. It would particularly be interesting to decipher whether these internalization events trigger local inflammation in response to the detection of the bacteria by the innate immune system, or promote the translocation of *S. warneri* across the intestinal barrier, thereby facilitating infection in deeper tissues. Another possibility would be that the internalization of *S. warneri* by intestinal cells constitutes a yet uncharacterized type of mutualistic interaction between human cells and gut bacteria.

## Conclusions

Our results highlight that some bacteria that are natural members of the human gut microbiota but not classified as pathogenic microbes, can invade intestinal cells. This work challenges the classic dichotomic conception of host–bacteria interactions in the gut, with commensal/symbiotic bacteria on the one hand, and *bona fide* pathogens on the other hand. This work paves the way for future studies aimed at deciphering the consequences of these internalization events on intestinal physiology, under both healthy and pathological conditions.

## Methods

### Preparation of mouse cecal contents

Eight-week-old male C57BL/6JRj mice (Janvier Labs, Le-Genest-Saint-Isle, France) were housed at 23 °C (5 animals/cage) with a 12-h light–dark cycle in regular open cages. All animals were fed a non-sterilized standard rodent diet (3430.PM.S10, Serlab, France). Drinking water was not sterilized. After 1 week of acclimatization to the animal facility, the animals were euthanized by an intraperitoneal injection of an overdose of ketamine (200 mg/kg BW) and xylazine (20 mg/kg BW). The caeca were then removed and opened longitudinally to recover the cecal contents.

### Cell culture

Human intestinal Caco2 cells (ATCC HTB-37) and human nonintestinal HeLa cells (ATCC CCL-2) were cultivated in Minimum Essential Medium Eagle (MEM; Eurobio) supplemented with 10% fetal calf serum (FCS), 2 mM l-glutamine, 1% nonessential amino acids (Sigma‒Aldrich) and 1 mM sodium pyruvate (Gibco). Human intestinal T84 cells (ATCC CCL-248) were cultivated in Dulbecco’s Modified Eagle’s Medium (DMEM)/F12 (1:1) (Eurobio) supplemented with 10% FCS and 2.5 mM l-glutamine. Murine RAW264.7 macrophages (ATCC TIB-71) were cultivated in DMEM supplemented with 10% FCS and 2 mM l-glutamine. All cell lines were cultivated in the presence of penicillin (1.10^4^ U/mL) and streptomycin (10 mg/mL) (Panbiotech), except during invasion assays.

### Bacterial strains

The bacterial strains used in this study were: *Staphylococcus warneri* AW25 type strain (DSM 20316, DSMZ, Germany), *Staphylococcus warneri* BOD010 strain (isolated from mouse cecal content; this study), *Staphylococcus aureus* RN4220 strain (DSM 26309, DSMZ, Germany), *Escherichia coli* K12 strain and *E. coli* transformed with the pRI203 plasmid encoding the *Yersinia pseudotuberculosis inv* locus (BUG1966; a kind gift from P. Cossart, Institut Pasteur, Paris, France) [20]. Staphylococcal species were grown in brain heart infusion (BHI) broth or agar plates (BD Biosciences) at 37 °C. *Escherichia coli* was grown in LB broth or agar plates (BD Biosciences). All bacterial strains used in this study were sensitive to 50 µg/mL gentamicin and 50 µg/mL imipenem.

### Invasion assays

Caco2, T84 and HeLa were seeded at a density of 1.6 × 10^5^ cells per cm^2^ and RAW264.7 cells were seeded at a density of 1 × 10^5^ cells per cm^2^ the day before invasion assays. Cells were cultivated in FCS- and antibiotic-free culture media, with or without 1 µg/mL cytochalasin D (Sigma‒Aldrich) for 1 h before the addition of bacteria. Bacteria grown in the exponential or stationary phases were washed twice with phosphate-buffered saline (PBS), added to cells at a MOI of 10 to 1000 and centrifuged on the cells for 3 min at 80×*g*. After 1 h of coincubation, the cells were washed twice with 1 × PBS and incubated with FCS-containing fresh medium supplemented with 50 µg/mL gentamicin (Euromedex) and 50 µg/mL imipenem (Sigma‒Aldrich) to kill extracellular bacteria. Two hours, 24 h or 48 h after the coincubation began, the cells were washed three times with 1 × PBS and lysed with PBS–0.2% Triton X-100 (Sigma‒Aldrich). The number of viable intracellular bacteria released from the lysed cells was assessed by plating cell lysates on agar plates.

### Transmission electron microscopy

Caco2 cells coincubated for 1 h with *S. warneri* and 1 h with gentamicin and imipenem were harvested using trypsin. A total of 8.4 × 10^5^ cells were centrifuged for 5 min at 1500×g and then resuspended in 100 µL of 2% w/v low gelling temperature agarose (2-hydroxyethyl agarose; Sigma‒Aldrich) in 0.1 M sodium cacodylate buffer (pH 7.2), incubated for 10 min on ice to solidify and cut into 1 mm blocks. Agarose blocks containing cells were then fixed for 2 h in 2% paraformaldehyde and 2% glutaraldehyde mixture (v/v) in 0.1 M sodium cacodylate buffer (pH 7.2) and washed (2 × 10 min) with cacodylate buffer. The samples were postfixed for 1 h in 1% osmium tetroxide (v/v) in cacodylate buffer and washed as previously described. Then, the samples were dehydrated in an ethanol series (50, 75, 95 and 2 × 100%) for 20 min each and transferred to an embedding resin (Spurr Resin) through a Spurr–ethanol series (8 h 25%, 16 h 50%, 8 h 75% and 2 × 24 h 100%). The resin was finally polymerized at 50 °C for 24 h. Ultrathin sections from the resin blocks (90 nm; EM UC6 Leica microsystems) were collected on coated formvar/carbon nickel grids and stained with uranyl acetate (0.5% w/v in methanol, 10 min) and Reynolds lead citrate (20 min). Observations of at least 190 cells from two independent resin blocks were made with an FEI Tecnai 12 Biotwin transmission electron microscope operating at 80 kV, with an ES500 W Erlangshen CCD camera (Gatan).

### Quantification of *S. warneri* in human fecal samples by quantitative PCR

DNA was extracted from the cecal contents of three independent groups of 8-week-old male C57Bl/6JRj mice (Janvier Labs, Le-Genest-Saint-Isle, France) or from human fecal samples from a biocollection of healthy individuals. Samples from 7 men (26–51 years old (mean 35.3); BMI: 21.6–24.8 kg m^−2^ (mean 23.7)) and 29 women (23–59 years old (mean 32.6); BMI: 19.1–24.5 kg m^−2^ (mean 21.4)) were included for analysis. DNA was extracted using the QIAamp DNA Stool Mini Kit (QIAGEN), including a bead-beating step (0.1 mm zirconia silica beads, BioSpec products, Bartlesville, USA) [40]. Quantitative real-time polymerase chain reaction (qPCR) was performed on these DNA samples using Mastercycler *ep* Realplex system (Eppendorf, Hamburg, Germany) and Itaq Universal SYBR Green Supermix (Bio-Rad). The primers used for the detection of *S. warneri* were Sw_sodA_F (5′-TCTGATATTCAAACTGCAGTAAGA-3′) and Sw_sodA_R (5′-AACAACTAACCAAGCCCAAC-3′). The specificity of the *S. warneri sodA* primer pair was evaluated by cloning the qPCR amplification products from at least three independent DNA samples, using the Zero Blunt PCR cloning kit (Invitrogen), and by verifying that more than 80% of the PCR products had a sequence corresponding to the *S. warneri sodA* gene. The primers used for the detection of Eubacteria were Eub-338F (5′-ACTCCTACGGGAGGCAGCAG-3′) and Eub-518R (5′-ATTACCGCGGCTGCTGG-3′) [41]. All samples were run in duplicate in 96-well reaction plates. Final concentrations were as follows: DNA, 4 ng/µL; primers 0.5 µM; SYBR Green Supermix 1X. The thermocycling conditions were as follows: initiation step 5 min at 95 °C; cycling stage: 5 s at 95 °C, 30 s at 63 °C or 60 °C (for *S. warneri* and Eubacteria primers, respectively), 40 cycles; melt curve stage 15 s at 95 °C, 15 s at 65 °C, increment of 1 °C every 10 s until reaching 95 °C. Quantification was performed by comparing the Cq of each sample with a standard curve made by diluting genomic DNA extracted from pure cultures of *S. warneri* and *E. coli,* for which cell counts were determined prior to DNA isolation. Non‐template controls were included on each plate.

### Statistical analysis

Comparison of *S. warneri* prevalence between males and females was performed using Fisher’s exact test. Correlations between *S. warneri* prevalence and age or BMI were evaluated using Spearman correlation coefficients. Comparisons of the internalization efficiencies of different bacterial species or under different cell culture conditions were performed using Friedman’s test with Dunn’s correction or with two-tailed Wilcoxon’s test. Comparisons of the internalization efficiencies of the bacteria in different growth phases were performed using the two-tailed Mann‒Whitney test. The kinetics of intracellular bacteria survival and MOI-dependent internalization were analyzed by 2-way ANOVA with Sidak’s correction. Statistical analyses were performed with GraphPad Prism 8 (GraphPad Software, San Diego, USA).

## Data Availability

All data generated or analyzed during this study are included in this published article.
